# Supplementary figures and images for: The expression of equine keratins K42 and K124 is restricted to the hoof epidermal lamellae of Equus caballus
Source: PLoS One. 2019 Sep 24;14(9):e0219234. doi: 10.1371/journal.pone.0219234 (PMC6759161; doi:10.1371/journal.pone.0219234)

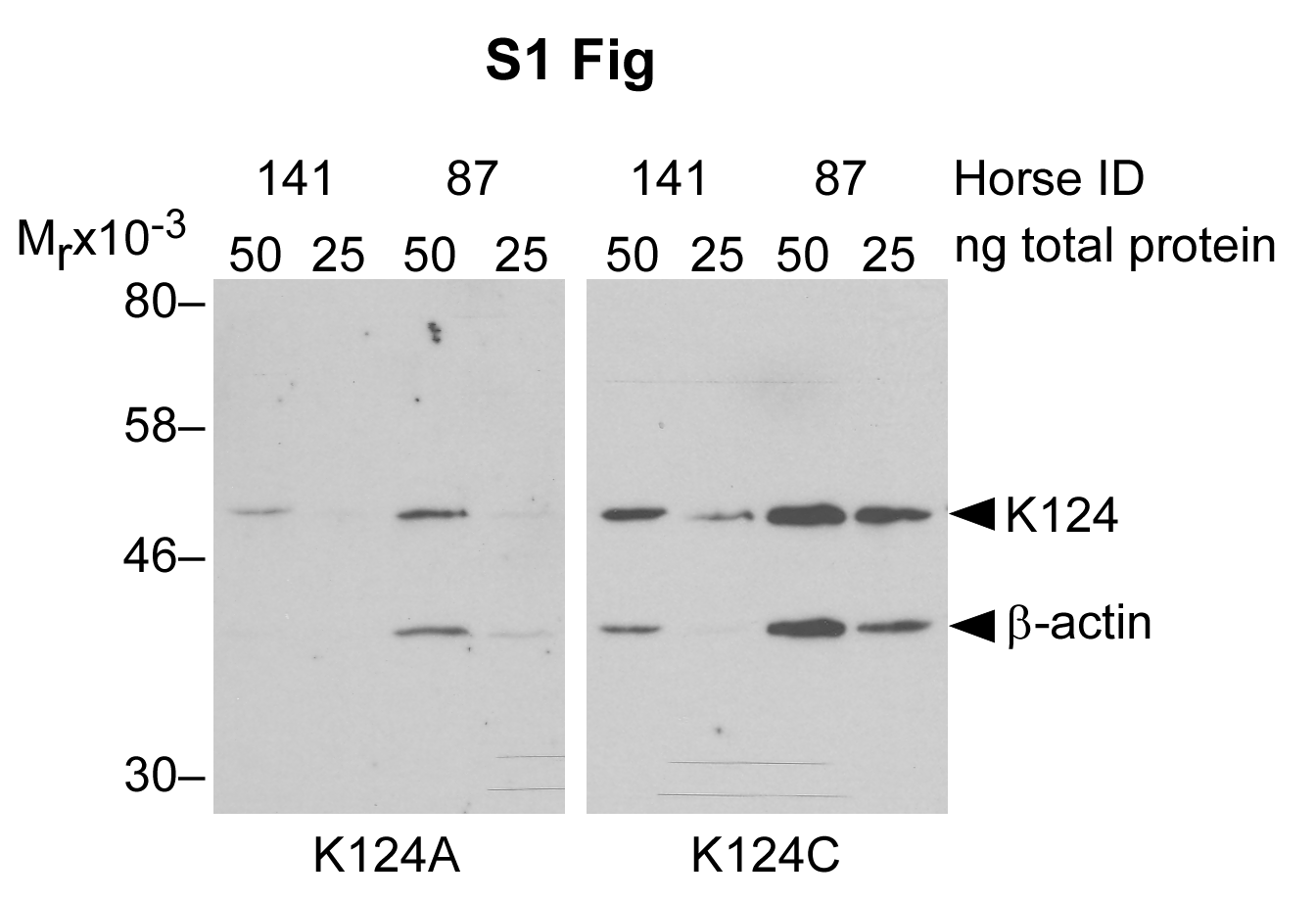

Supplement: S1 Fig — Representative immunoblots using affinity-purified anti-K124 mAb, clones A (left) and C (right) at 1:5,000 dilution. Total protein extracted from lamellar tissue from three horses (two shown, as indicated above blots) loaded at 50 ng and 25 ng per lane (as indicated above blots). K124 and β-actin immunoblotting and detection were performed as described in the Methods section with the exception that chemiluminescence detection was performed using 2.5 mM luminol and 390 μM p-coumaric acid (5x strength). K124A and K124C are immunoreactive with a 54 kDa relative molecular mass (Mr) band, which is clearly visible at 50 ng and 25 ng total protein load for K124C and 50 ng protein load for K124A. (TIF) [file pone.0219234.s001.tif]

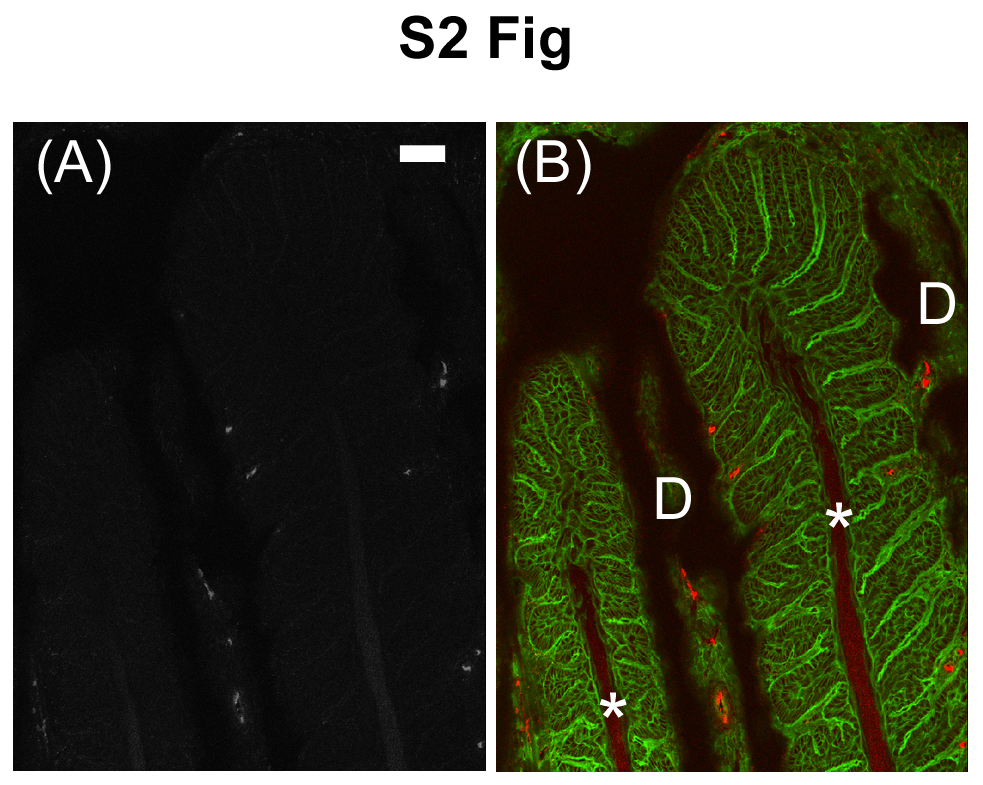

Supplement: S2 Fig — Lamellar tissue cryosection, serial to the one shown in Fig 6, subjected to indirect immunofluorescence and fluorescein-conjugated wheat germ agglutinin (WGA) as a counterstain, omitting the K124C mAb to show non-specific staining (n = 3 using samples from 3 horses, representative image shown). (A) Red channel, secondary antibody alone (white). (B) Secondary antibody alone (red) and fluorescein-WGA counterstain (green). Scale bar = 20 μm. Images were collected and adjusted under settings identical to those applied to Fig 6. (TIF) [file pone.0219234.s002.tif]
