# Supplementary material for: The expression of equine keratins K42 and K124 is restricted to the hoof epidermal lamellae of Equus caballus
Source: PLoS One. 2019 Sep 24;14(9):e0219234. doi: 10.1371/journal.pone.0219234 (PMC6759161; doi:10.1371/journal.pone.0219234)
Supplement: S1 Table — (PDF) [file pone.0219234.s003.pdf]

S1 Table. Breed, age, and sex of horses (*E. caballus*) used in experiments.

Horse ID#	Breed†	Age (years)	Sex‡
**28**	QH	10	MC
**29**	TB	24	F
**48**	TB	2	MC
**50**	TB	7	MC
**52**	TB	17	MC
**57**	WB	6	MC
**61**	WB	11	MC
**70**	TB	11	F
**84**	TB	2	MC
**87**	ASB	20	MC
**88**	TB	4	F
**91**	PF	14	F
**92**	WB	18	F
**93**	WP/TB	13	MC
**98**	QH/Ar	13	MC
**101**	MO	13	MC
**102**	QH	17	MC
**106**	TB	3	F
**108**	PO	2	F
**111**	TB	18	F
**113**	TB	12	F
**114**	TB	12	F
**127**	TB	3	MC
**129**	Ar	22	F
**130**	TB	4	MC
**132**	QH	6	MC
**140**	TB/WB	14	F
**141**	RMH	21	F
**143**	TB	3	M
**144**	TB	5	MC
**145**	WB	4	MC
**148**	TB	3	F
**163**	TB	3	F
**168**	QHX	9	MC
**169**	TB	20	MC
**170**	TB	5	F
**171**	TB	6	MC

†Breed abbreviations: QH: American Quarter Horse; TB: Thoroughbred; WB: Warmblood; ASB: American Saddlebred; PF: Paso Fino; WP/TB: Welsh pony/TB cross; QH/Ar: American Quarter Horse/Arabian cross; MO: Morgan; PO: Pony; Ar: Arabian; TB/WB: Thoroughbred/Warmblood cross; RMH: Rocky Mountain Horse; QHX: American Quarter Horse cross.

‡Sex abbreviations: F: female; M: male; MC: castrated male
