# Supplementary material for: The expression of equine keratins K42 and K124 is restricted to the hoof epidermal lamellae of Equus caballus
Source: PLoS One. 2019 Sep 24;14(9):e0219234. doi: 10.1371/journal.pone.0219234 (PMC6759161; doi:10.1371/journal.pone.0219234)
Supplement: S2 Table — (PDF) [file pone.0219234.s004.pdf]

S2 Table. Horses (*E. caballus*) used for each experiment

Experiment[†]	Tissue	Horse[‡]
RT-PCR	Lamellar	70, 84, 101, 102, 111, 145
	Haired Skin	102, 108, 111, 145
	Corneal Limbus	88, 92, 98
	Hoof Coronet	113, 114, 144
ISH	Lamellar	84, 111, 127, 140, 163
	Haired Skin	28, 29, 113, 114
	Hoof Coronet	113, 114, 163
IB	Lamellar	87, 114, 129, 130, 132, 141, 148
	Haired Skin	50, 52, 57, 61
	Hoof Coronet	50, 168, 169, 170
	Corneal Limbus	91, 92, 93
	Chestnut	168, 169, 170
	Tongue	168, 169, 170
	Oral Mucosa	168, 169, 170
	Unhaired (Glabrous) Skin	168, 169, 171
IIF	Lamellar	106, 130, 143, 163
	Haired Skin	48, 130, 148
	Hoof Coronet	130, 144, 163

[†]Experiment abbreviations: RT-PCR: reverse transcriptase-polymerase chain reaction; ISH: in situ hybridization; IB: protein immunoblotting; IIF: indirect immunofluorescence histology

[‡]See S1 Table for age, breed, and sex of individual horses.
